# Interactive Micromanipulation of Picking and Placement of Nonconductive Microsphere in Scanning Electron Microscope

**DOI:** 10.3390/mi8080257

**Published:** 2017-08-21

**Authors:** Ning Cao, Shaorong Xie, Zhizheng Wu, Mei Liu, Hengyu Li, Huayan Pu, Jun Luo, Zhenbang Gong

**Affiliations:** School of Mechatronic Engineering and Automation, Shanghai University, Shanghai 200444, China; zhizhengwu@shu.edu.cn (Z.W.); mliu@shu.edu.cn (M.L.); lihengyu@shu.edu.cn (H.L.); phygood_2001@shu.edu.cn (H.P.); luojun@shu.edu.cn (J.L.); nflcsh@gmail.com (Z.G.)

**Keywords:** interactive micromanipulation, contact detection, picking and placement, nonconductive microsphere

## Abstract

In this paper, classified theoretical models, consisting of contact with and placement of microsphere and picking operations, are simplified and established to depict the interactive behaviors of external and internal forces in pushing manipulations, respectively. Sliding and/or rolling cases, resulting in the acceleration of micromanipulations, are discussed in detail. Effective contact detection is achieved by combining alterations of light-shadow and relative movement displacement between the tip-sphere. Picking operations are investigated by typical interactive positions and different end tilt angles. Placements are realized by adjusting the proper end tilt angles. These were separately conducted to explore the interactive operations of nonconductive glass microspheres in a scanning electron microscope. The experimental results demonstrate that the proposed contact detection method can efficiently protect the end-tip from damage, regardless of operator skills in initial positioning operations. E-beam irradiation onto different interactive positions with end tilt angles can be utilized to pick up microspheres without bending the end-tip. In addition, the results of releasing deviations away from the pre-setting point were utilized to verify the effectiveness of the placement tilt angles.

## 1. Introduction

Micro/nano manipulation, fabrication, and assembly techniques have played significant roles in constructing integrated circuits, micro/nano sensors [1,2], and micro electromechanical systems (MEMS) [3,4,5,6], where manipulation is the most crucial factor [7]. Integrated MEMS end-effectors, possessing accurate position actuators and force sense feedback, have been widely used in micro/nano manipulation [8,9,10,11]. Hence, designing proper actuators and sensing units in order to match device functions is required to further explore interactive behaviors, leading to system improvements.

Scheduling interactive manipulations can be, to some extent, decided by operating conditions and objects. For instance, the manipulation of soft biological cells needs to be determined by operation orientations and the right amount of force [12]. However, the manipulation of micro/nano particles has become a highly effective method for investigating mobility and the further determination of micro/nano mechanics and/or tribology [13]. Presently, most studies mainly focus on conductive sphere-like or sphere objects, such as sphere-like silver nanoparticles [13], gold nanoparticles [14], and tungsten particles [15], instead of nonconductive objects, in general. Thus, this will result in a lack of relevant force analyses and interactive modes for designing integrated build-in actuators and/or sensors to manipulate nonconductive objects. Additionally, essential characteristics of nonconductive objects may exhibit unique properties due to special physical differences. Nonconductive micro/nanosphere-like particles can be studied to enhance the properties of micro/nano operating devices, which also play guidance roles in manipulating non-sphere-shaped objects.

Compared with the manipulation of nanoparticles carried out using conventional or modified atomic force microscope (AFM) systems [16,17,18], operations in scanning electron microscopes (SEMs) exhibit the distinct advantages of a large field of view and real-time, high capture capability. However, a limitation of the imaging principle is that it is not likely to automatically obtain the depth in the *Z*-direction [19]. Therefore, determination of the depth value is the foundation of achieving three-dimensional operations, while the interactive contact detection, regarded as the initial positioning, is usually used to judge whether it will continue to accomplish highly effective and quantitative manipulations. With respect to contact detection methods, there are some studies that examined direct touch sensors [8,20], the depth from focus method [21], vision-based sliding detection [22,23], and shadow-based depth detection [24], which have been adopted to enhance the degree of automatic micro/nano manipulation in SEMs. However, a low moving speed and long consumption of time will restrict high speeds and a reliable automatic operation. In addition, it may directly bend the end-effector tip and the soft operated object. Therefore, there is a demand for the pursuit of a convenient, highly efficient, and low-cost contact method, avoiding damage to the end-effector tip for automatic operations of sphere-like or other shaped objects.

In the interactive manipulation of micro/nano objects, picking up and placement behaviors are considered as the main challenges as microscale adhesion forces dominate and generally need to be overcome [25]. Researchers have studied this using different methods, e.g., electrostatic forces [15,26,27] or capillary forces [14], pushing or pulling nanoparticles [28], gripper-like dual AFM cantilever tips [16], and micro/nano mechanical grippers [29,30,31,32]. However, to deal with objects of different shapes, the operation of end-effectors is mainly focused on the interaction results of picking and placement. Few interactive methods that combine different positions and end tilt angles have been quantitatively taken into account in micro/nano manipulation, while the influence of beam irradiation on locations have generally been ignored. In view of the limitations of operation space, a single end can allow for complex operations compared with a bulk end-effector. Consequently, a deeper depiction of the interactive manipulations existing in the procedures of mechanical contact, picking, and placement of micro/nano objects is required.

In this paper, theoretical interactive models consisting of contact, picking, and placement of microspheres are simplified, established, and discussed. Microspheres are classified into two types of different mechanical operations. Interactive experiments were conducted on glass microspheres using a scanning electron microscope, and are discussed separately in detail. An interactive detection, combining the distinct alterations of light-shadow with relative movement displacement between the tip-sphere interface, was utilized to realize real contact. The manipulation of methods, consisting of typical interactive positions and different end tilt angles, were conducted to study the effects of damaging the adhesion interface and picking behaviors. Moreover, the influence of e-beam irradiation locations on electrostatic picking is discussed. Finally, the proper end tile angles and releasing results are discussed in terms of placement operations.

## 2. Theoretical Modeling Analysis

**Figure 1:**
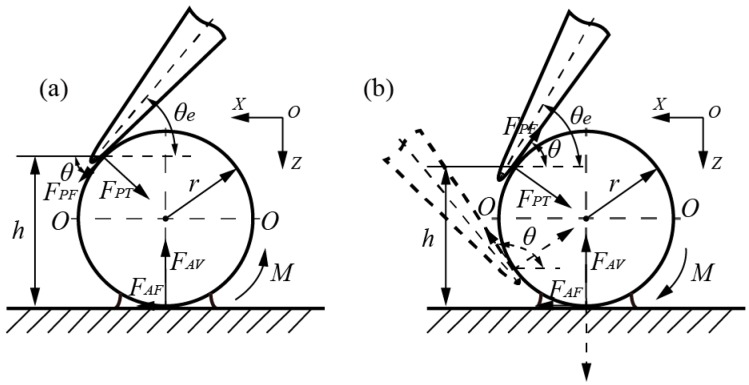
Simplified theoretical models show the interactive forces and moments during pushing micromanipulation, resulting in sliding and/or rolling behaviors, based on the coupled roles of active forces and adhesion forces; (**a**) interactive contact or releasing operation above the central plane *O*–*O* of a microsphere; and (**b**) picking operations are divided into two cases, consisting of locations above and beneath the central plane *O*–*O* of a microsphere. Notes: In the plane of *X*–*Z*, *θ_e_* is the end tilt angle, *θ* is the angle formed by the active friction force *F_PF_* and the direction of *X*, *M_roll_* is the active moment, and *h* is vertical height from the interactive position to the substrate.

To deeply investigate the interactive relationships (located in the tip-sphere and/or sphere-substrate) during the manipulation of microspheres, classified theoretical models were simplified, as shown in Figure 1, to show interactive forces and moments in pushing operations, which may result in sliding and/or rolling behaviors, based on the coupled roles of active forces and adhesion forces. Both active forces from the end-tip and adhesive forces from the adhesive layer interface are decomposed into components of the normal force and friction force. Specifically, there are four types of force components: Applied active friction force *F_PF_* and normal force *F_PT_* from the end-tip; and friction force *F_AF_* and normal force *F_AV_* existing in the adhesive interface. In addition, some assumptions are made: (a) The end-tip wear is not considered in manipulation; (b) the geometrical shape and size of spheres are uniform; (c) the substrate surface is flat enough to manipulate microspheres. In this study, interactive manipulations mainly focus on the procedures of contact detection, picking, and placement of microspheres, which are generally divided into two models, according to different mechanical operations.

### 2.1. Adhesive Interface Forces

Adhesive interface forces from the tip–sphere or sphere–substrate layer can be regarded as a combination of electrostatic forces, van der Waals forces, and capillary forces [33]. Based on the contact mechanics model established by Piétrement [34], the maximum adhesive force in the adhesion layer can be approximated as follows:

(1) $$F_{A}=\pi R_{A}\gamma_{A}\left(0.267\alpha_{A}^{2}-0.767\alpha_{A}+2\right)$$

where *R_A_* and *γ_A_* are the equivalent radius and effective surface energy of interface, respectively [35]; the parameter *α_A_* = (1 − *e*^−^^1.157(*R*_A_γA 2/*K*^2^d0A 3)^1/3^/0.913^)/1.018; *d*_0*A*_ is the interatomic equilibrium spacing [28]; effective elastic modulus *K* = (4/3)((1 − $v_{1}^{2\;}$)/*E*_1_ + (1 − $v_{2}^{2\;}$)/*E_2_*)^−^^1^, where *ν*_1_ and *ν*_2_ are the Poisson ratios of interaction mediums, while *E*_1_ and *E*_2_ are those of Young′s moduli, respectively.

The friction forces existing in the adhesive interface can be calculated as follows:

(2) $$F_{AF}=\pi \tau_{A}{\left(R_{A}F_{AV}/K\right)}^{2/3}$$

where *τ_A_* is interfacial shear strength.

The maximum resistance moment *M*_max_is expressed by [28]:

(3) $$M_{\max}=6\pi R_{A}\gamma_{A}\xi$$

where *ξ* is the contact radius.

### 2.2. Interactive Contact or Releasing Operation

The theoretical model in Figure 1a illustrates the force analysis when the end-tip makes contact with the microsphere. As end tilt angle *θ_e_* is an acute angle, angle *θ* is equal to end tilt angle *θ_e_*, with the addition of point contact in the tip-sphere interface. Essentially, *θ_e_* ≈ *θ*, where *θ* is the angle formed by active friction force *F_PF_* and the positive direction of *X*.

Once the end-tip has made contact with the microsphere, by moving it downward along the *Z*-direction, the rolling sphere may be approximated by:

(4) $$F_{PF\max}-F_{AV}\sin\theta +F_{AF}\cos\theta \leq 0$$

(5) $$M_{\max}-M_{roll}\leq 0$$

where *F_AV_* and *F_AF_* are components of normal force and friction force, derived from the sphere–substrate adhesion interface, respectively; *F*_*PF*max_ is the maximum active friction force from the end.

The active moment (*M_roll_*) from the end-tip required to roll the sphere is expressed by:

(6) $$\begin{array}{l} M_{roll}=\left(F_{PT}\sin\theta -F_{PF}\cos\theta \right)h-\left(F_{PT}\cos\theta +F_{PF}\sin\theta \right)\left(h-r\right)\tan\theta \\ \;=r\left(F_{PT}\sin\theta -F_{PF}\cos\theta \right)\left(1+\cos\theta \right)-r\left(F_{PT}\cos\theta +F_{PF}\sin\theta \right)\sin\theta \end{array}$$

where *F_PT_* is the active normal force from the end; *h* is the vertical height from the interactive position to the substrate, representative of the arm of forces (shown in Figure 1).

In other words, the end tilt angle of *θ_e_* is required to simultaneously satisfy the equations, as follows:

(7) $$\theta_{e}=\theta \geq \arcsin\left(\left(F_{PF\max}F_{AV}+F_{AF}\sqrt{F_{AV}^{2}-F_{PF\max}^{2}+F_{AF}^{2}}\right)/\left(F_{AV}^{2}+F_{AF}^{2}\right)\right)$$

(8) $$\begin{array}{l} \theta_{e}=\theta \geq \arcsin((\sqrt{F_{PT}^{2}{\left(rF_{PF}+6\pi R_{A}\gamma_{A}\xi \right)}^{2}-\left({\left(6\pi R_{A}\gamma_{A}\xi \right)}^{2}+2rF_{PF}\cdot 6\pi R_{A}\gamma_{A}\xi \right)\left(F_{PT}^{2}+F_{PF}^{2}\right)}\\ +F_{PT}\left(rF_{PF}+6\pi R_{A}\gamma_{A}\xi \right))/r\left(F_{PT}^{2}+F_{PF}^{2}\right)) \end{array}$$

Alternatively, there is no rolling case, whereas the end-tip may slide onto the sphere; thus, end tilt angle *θ_e_* is required to be the opposite of that of Equations (7) and (8).

If a microsphere stuck onto the end-tip needs to be released onto the substrate, it will damage the adhesive tip-sphere interface in the first step of placement operations. Similarly, it should be noted that it is possible to generate relative sliding after damaging the adhesion layer. Therefore, properly adjusting end tilt angle *θ_e_* is also required to meet conditions, which are opposite to those of Equations (7) and (8).

### 2.3. Interactive Picking Operation

Picking operations are divided into two cases, consisting of locations above and beneath the central plane (*O*–*O*) of a microsphere, as shown in Figure 1b. Once the interactive position located above the central plane is determined, both the normal and friction force components can stay in their original directions, despite end tilt angle *θ_e_* being an acute angle or obtuse angle. Essentially, angle *θ* is the constant angle formed by the active friction force *F_PF_* and the negative direction of *X*. When the interactive position is located beneath the central plane, obtuse angle *θ* is also decided by the interactive positions, and, in this way, end tilt angle *θ_e_* must be an obtuse angle, which is always larger than the obtuse angle of *θ*.

(A) Assuming the picking positions are at the left side of the sphere, active friction force *F_PF_* is presented along the negative *Z*-direction and active moment *M_roll_* is exhibited along a clockwise direction. If the interactive positions are located above central plane *O*–*O*, the rolling sphere appears in accordance with the formulas below:

(9) $$F_{PF\max}+F_{AV}\sin\theta -F_{AF}\cos\theta \leq 0$$

(10) $$M_{\max}-M_{roll}\leq 0$$

where active moment *M_roll_* can be expressed by:

(11) $$\begin{array}{l} M_{roll}=\left(F_{PT}\sin\theta +F_{PF}\cos\theta \right)h-\left(F_{PT}\cos\theta -F_{PF}\sin\theta \right)\left(h-r\right)\tan\theta \\ \;=r\left(F_{PT}\sin\theta +F_{PF}\cos\theta \right)\left(1+\cos\theta \right)-r\left(F_{PT}\cos\theta -F_{PF}\sin\theta \right)\sin\theta \end{array}$$

Therefore, the *θ* required can be satisfied by the formulas that follow:

(12) $$\theta \leq \arcsin\left(\left(F_{AF}\sqrt{F_{AF}^{2}-F_{PF\max}^{2}+F_{AV}^{2}}-F_{PF\max}F_{AV}\right)/\left(F_{AF}^{2}+F_{AV}^{2}\right)\right)$$

(13) $$\begin{array}{l} \theta \leq \arcsin((\sqrt{F_{PT}^{2}{\left(6\pi R_{A}\gamma_{A}\xi -rF_{PF}\right)}^{2}+\left(2rF_{PF}\cdot 6\pi R_{A}\gamma_{A}\xi -{\left(6\pi R_{A}\gamma_{A}\xi \right)}^{2}\right)\left(F_{PT}^{2}+F_{PF}^{2}\right)}\\ +F_{PT}\left(6\pi R_{A}\gamma_{A}\xi -rF_{PF}\right))/r\left(F_{PT}^{2}+F_{PF}^{2}\right)) \end{array}$$

Alternatively, in addition to meeting the relationship of *M*_max_ ≥ *M_roll_*, the pure sliding sphere is also required to satisfy the formula that follows:

(14) $$F_{AF\max}-F_{PT}\sin\theta -F_{PF}\cos\theta \leq 0$$

Therefore, *θ* can be expressed by:

(15) $$\theta \leq \arcsin\left(\left(F_{AF\max}F_{PT}+F_{PF}\sqrt{F_{PF}^{2}-F_{AF\max}^{2}+F_{PT}^{2}}\right)/\left(F_{PF}^{2}+F_{PT}^{2}\right)\right)$$

(B) When interactive positions are located underneath central plane *O*–*O*, normal force *F_AV_*, the imaginary line, presents along the *Z*-direction, as shown in Figure 1b. The rolling sphere can be generated if the active moment is greater than that of the maximum resistance, similar to Equation (10). At the same time, active friction force *F_PF_* should meet the formula that follows:

(16) $$F_{PF\max}-F_{AV}\sin\theta -F_{AF}\cos\theta \leq 0$$

Therefore, angle *θ* is expressed by:

(17) $$\theta \leq \arcsin\left(\left(F_{AF}\sqrt{F_{AF}^{2}-F_{PF\max}^{2}+F_{AV}^{2}}+F_{PF\max}F_{AV}\right)/\left(F_{AF}^{2}+F_{AV}^{2}\right)\right)$$

Similarly, the case where a sliding sphere may occur can be interpreted using Equation (14); however, it is noted that the obtuse angle of *θ* is used in calculations. Meanwhile, it should meet the relationship of *M_max_* ≥ *M_roll_* in the pure sliding sphere.

Due to the point contact between the end-tip arc contour and the sphere, interactive picking manipulation behaviors can be affected by the active friction component angle of *θ* and different contact positions. Once the vertical height *h* is determined, the different end tilt angle, *θ_e_*, will merely affect the values of the active force components, which possess constant directions.

## 3. Interactive Manipulation Experiments and Discussion

**Figure 2:**
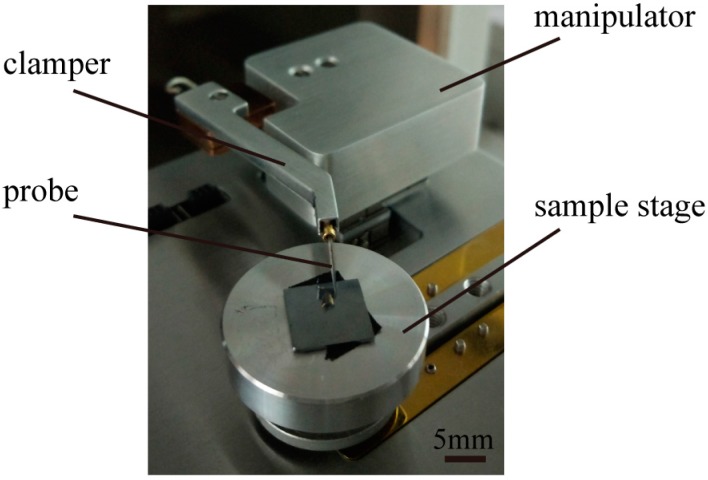
Experimental setup, consisting of a micro/nano manipulator clamping a flexible end probe, embedded in an SEM vacuum chamber.

As shown in Figure 2, the experimental setup consists of a micro/nano manipulator, which possesses coarse and fine modes with three degrees of freedom (DOF) that switch in real time according to operation tasks, embedded in a scanning electron microscope (SU3500, Hitachi, Japan). The coarse positioner (SL0610, SmarAct, Oldenburg, Germany) and fine piezoelectric stage (AE0203D04F, Thorlabs, Newton, NJ, USA) have a travel of 10 mm, with a 100-nm resolution, and a travel of 20 μm with a 1-nm resolution, respectively. A single tungsten probe with a stiffness of 0.175 N/m, which was treated as a flexible end-effector calibrated by the commercial AFM cantilever, was mounted on the manipulator clamper. Experiments used a glass microsphere with a diameter of 10 μm as representative of nonconductive microspheres.

Micromanipulation experiments studying interactive behaviors primarily included contact detection, picking, and releasing operations. Coarse motion with an open loop control was used in contact detection, while fine motion with a position sensor, to realize closed loop control, was adopted in picking-and-placing operations.

Concerning experimental conditions, Young’s moduli and Poisson ratios of the sphere, substrate, and probe were 55 Gpa, 187 Gpa, and 130 Gpa, and 0.25, 0.27, and 0.35, respectively. In particular, we assumed the maximum friction factors in the sphere-substrate and tip-sphere interfaces to be 0.6 and 0.2, respectively.

Assuming a limited consumption of operation time, adhesive forces from the new interface, formed using ultrashort beam irradiations, can be ignored in the theoretical analysis based on the experimental conditions. For instance, no adhesive forces between the tip-sphere layer were considered in contact and picking manipulations. Similarly, adhesive force is also handled in the sphere-substrate interface during placement operation.

In order to depict interactive manipulations, the theoretical analysis can be quantified to guide experiments. Obviously, real contact is an important premise in highly efficient pushing manipulations. When the end tilt angle is 0 < *θ_e_* < 22°, the rolling sphere cannot occur on the basis of theoretical formulas. In comparison, the end-tip may slide on the top sphere surface due to adhesive forces in the sphere-substrate interface. Otherwise, the microsphere can be rolled and the end-tip can slide when the tilt angle is 22° < *θ_e_* < 45°. Under an extreme case, a rolling sphere occurs ahead of end-tip sliding when the tilt angle is 45° < *θ_e_* < 90°.

A new adhesive layer between the tip and the sphere needs to be damaged to accomplish valid operation of sphere release, so the rolling sphere is the first step. Similarly, the microsphere is rolled when the end tilt angle is 0 < *θ_e_* < 30°.

Picking manipulations are divided into two cases, including the applied positions above and beneath the sphere central plane. Once an interactive position (i.e., vertical height *h*) is determined, the active friction angle (*θ*) can be uniquely obtained. When interactive positions are located in the upper section of the semi-sphere, a microsphere can always be rolled, regardless of the end tilt angle *θ_e_*. In particular, the end-tip may also slide onto the sphere when the active friction angle is *θ* < 26°. On the other hand, a rolling and/or sliding sphere may occur. When interactive positions are located in the lower section of the semi-sphere, a microsphere can generate rolling and/or sliding behaviors without end-tip sliding when the active friction angle is 90° < *θ* < 135°; otherwise, the microsphere can be rolled and the end-tip slides on the surface.

### 3.1. Contact Detection in Initial Positioning

**Figure 3:**
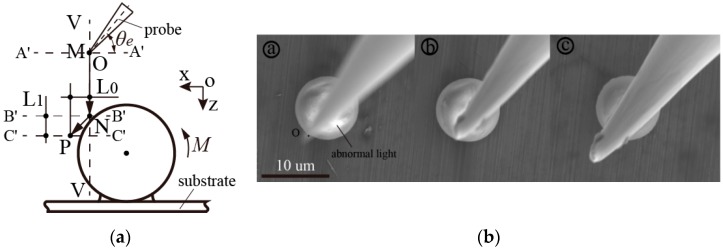
(**a**) Sketch map illustrating an end probe tip, having different tilt angles (*θ_e_*), which moved down vertically. This was produced by combining the relative movement displacement with distinct alternations of light-shadow to detect whether it had made contact with the operated sphere; (**b**) experimental photos ⓐ, ⓑ, and ⓒ; correspond with end-tip reference point *O*, located at point *M*, *N,* and *P,* in the *Z*-direction, respectively.

Before end probe picking operations, the generation of real contact with a sphere, as soon as possible in the initial positioning, is required. Figure 3 shows the procedures of the sketch map and experimental photos of contact detection, respectively. First, the selection of terminal point *O* of the end-tip, having a tilted angle of *θ_e_* as the reference point, was tracked in the experiments. Its arbitrary initial horizontal height of *A′–A′* is located above the uppermost part of the sphere surface. Following this, on the basis of abnormal light and alterations of light-shadow, the end-tip was moved down from initial point *M* to point *N* located at height *B′–B′*, along vertical line *V–V*. Following this, it is apparent that reference point *O* moved obliquely to point *P*, laid at a height of *C′–C′* under the circumstances of a rolling and/or sliding sphere.

Assuming that the geometrical shape of the end-tip and the end tilt angle *θ_e_* are determined, the vertical height difference from point *N* to the upper peak point of the sphere can be calculated. After the end-tip has made contact with the sphere, interactive behaviors occurred and relative movement displacement *L_0_* was also exhibited in the *X–Y* image plane. The relative vertical height *L_1_* can be theoretically calculated by the relative displacement *L_0_* and the end tilt angle *θ_e_*. Therefore, the relative vertical height of the end-tip from point *P* to the upper peak point of the sphere can be approximately obtained. After the real contact has been detected, highly efficient position initialization is completed to conduct the follow-up experiments.

Taking easy initial positioning and measurements into consideration, the outer contour of the microsphere in the *X–Y* plane was used as a reference boundary, being at the vertical line of *V–V*. An end tilt angle of *θ_e_*, from 10° to 70° in increments of 10 degrees, was adopted to study the interactive contact behaviors under a magnification of 4.5 × k.

Along with moving the end-tip down vertically, the extensive light sheltered by the end-tip was enhanced with a decrease in the *Z* height. In experimental procedures, it was seen that the abnormal light was always located on the top surface of a sphere, which was determined via charge accumulation from nonconductive characteristics. This had a more remarkable effect compared to that of a conductive object. Therefore, distinct alterations of light-shadow may be used to judge how close the end-tip is to the sphere. Essentially, this can be more easily utilized to approximately measure the relative *Z* height of the end-tip located above the sphere. In addition, quantitative analyses of the frequent area ratio of the light-shadow of nonconductive microspheres would be beneficial in improving operation efficiency.

Contact detection, prior to follow-up procedures, is the crucial initial positioning step in highly efficient and reliable operations. As point contact can be produced between the body of the end-tip and the top surface of a sphere, the end-tip will be effectively protected from damage. Thus, it is possible that the end-tip can be used to determine relative movement displacements within the *X*–*Y* plane using real-time imaging after generating real contact behavior.

**Figure 4:**
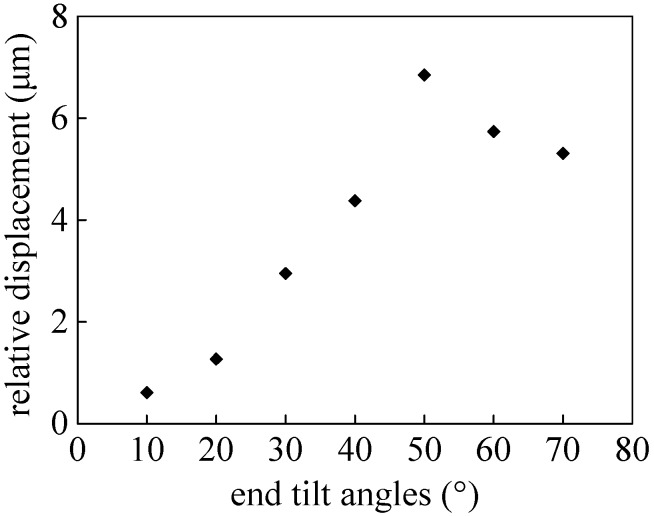
Relative movement displacement of *L_0_* against different end tilt angles using the contact detection method.

Relative movement displacements of *L_0_* against a variety of end tilt angles are shown in Figure 4. If the end tilt angles are too small, this will result in the tip sliding with only the deformation of the end. In contrast, excessively large end tilt angles can generate extreme movement, induced by the sphere rolling ahead of the tip sliding. It is demonstrated that end tilt angles within approximately 30°–40° can be considered as reasonable angles to accomplish contact detection, suitable for detection after momentary contact. These can be interpreted by the combined actions of rolling sphere and end-tip sliding, in accordance with the theoretical analysis. Hence, when coupled with light-shadow alterations, end-tip sliding and/or sphere rolling, contact detection can effectively protect the end-tip against damage, in order to improve efficiency, which possesses a prominent automatic ability regardless of operator skill in initial positioning manipulations.

### 3.2. Picking Interactive Manipulation

Picking interactive manipulations primarily include two steps: the first is the damaging of the sphere-substrate adhesive interface, and the second comprises picking operations utilizing e-beam irradiation between the tip-sphere interface.

**Figure 5:**
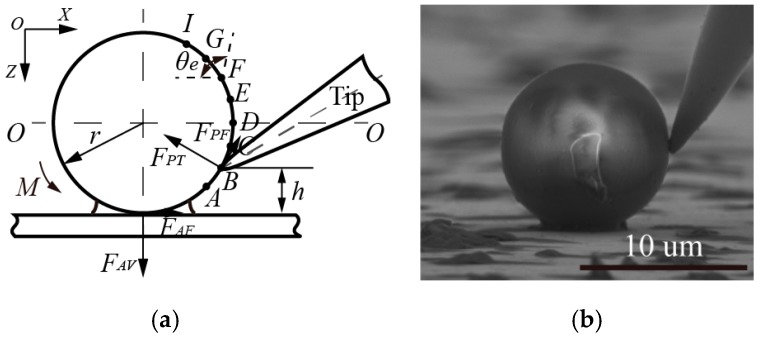
(**a**) Picking operations conducted using two different interactive methods: typical interactive positions and different end tilt angles, with increments of 15 degrees, were utilized to analyze the minimum required active forces and the interactive effects, respectively; and (**b**) experimental photo of the picking operation.

As shown in Figure 5, the influences of two different interactive methods for picking manipulations, including typical interactive positions and different end tilt angles, were examined to analyze picking interactive effects and to obtain the minimum required active forces. Considering the quantitative divisions and easy installation, counterclockwise increments of 15 degrees were adopted to position the typical interactive positions, from point *A* to point *I*, where point *D* is located on central plane *O*–*O*. Similarly, assuming that any interactive position was selected, minimum ultimate angle *θ_e_* will be *θ*. By uniformly increasing the increments by 15 degrees, maximum end tilt angle *θ_e_* should be restricted to 165°.

#### 3.2.1. Damaging Adhesive Interface

**Figure 6:**
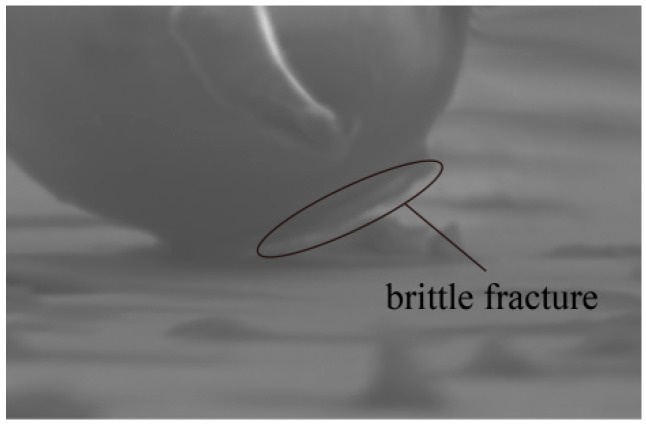
Photo exhibiting brittle fracture of the adherent interface formed between the sphere-substrate interface.

**Figure 7:**
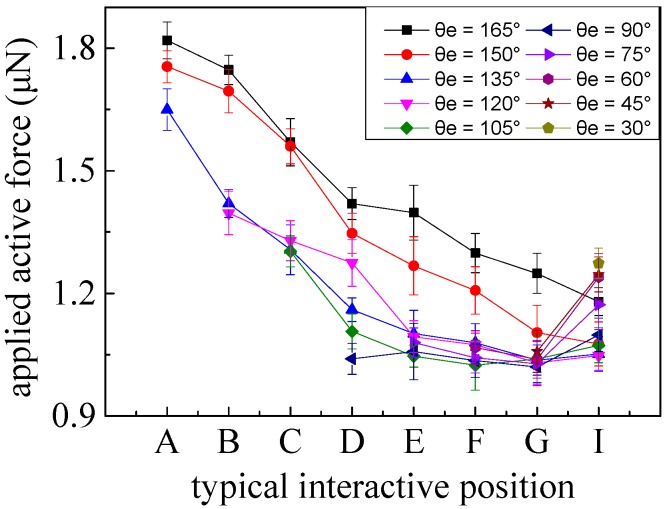
Minimum applied active forces, with errors, to overcome adhesion forces by coupling typical interactive positions with different end tilt angles.

The presence of the adhesive layers between both interfaces, sphere-substrate and tip-sphere, can be formed by the surface contaminants [36]. In particular, the adhesive layer in the sphere-substrate interface is dependent on the degrees of hydrophilia. Assuming the same operation conditions, a hydrophilic object has a bigger meniscus shape than that of a hydrophobic object. The adhesion force is related to the intrinsic properties of the interfacial system including the operated object and interface geometry. Because of the regular and uniform shape of the brittle fracture existing in nonconductive sphere-substrate contact arc areas, it can accelerate the failure of the adhesive interface to accomplish a rolling sphere, as shown in Figure 6. Also, the small smooth meniscus shape can be interpreted by the relatively hydrophobic property of the nonconductive sphere. Apparently, forces active enough to destroy the adhesion layer interface are required to overcome the adhesion forces. In particular, a time-dependent adhesive layer interface can affect the minimum active forces obtained uniformly in experiments, so the experimental conditions and time must be strictly controlled. Considering the interconnectedness of the elements being manipulated, the forces from coupling typical interactive positions with different end tilt angles are shown in Figure 7, respectively. Each curve indicates the minimum applied active forces obtained at typical interactive positions by keeping a certain tilt angle, with each error bar representing five trials.

Along with raising the height *h* of typical interactive positions, the applied active force basically had a declining trend, which may be explained by the prominent effect of vertical distance *h*, representative of a force arm in the active moment. For the same typical interactive position, it also basically decreased when the end tilt angle *θ_e_* decreased. When the interactive operations were located from contact point *D* to point *I*, they exhibited minor active forces. After the adhesive layer had been damaged, sphere rolling and/or sliding occurred, according with the theoretical predictions. However, once an obtuse angle *θ_e_* is adopted, it is highly likely to result in the damaging of the end-tip terminal when the active forces applied are not enough to overcome adhesive interface forces. Similarly, the interactive positions from *A* to *C*, with obtuse angles of *θ_e_*, were also imposed to roll the sphere (consistent with the theoretical analysis), which also protected the tip terminal. In particular, failure of the end-tip terminal, generated at position *A*, can be interpreted by the lower height (*h*). An interactive position located at point *I* with a tilt angle of 30° can generate a sliding case ahead of a rolling sphere, different from those of the theoretical analyses, due to model simplification.

#### 3.2.2. Picking Operations

**Figure 8:**
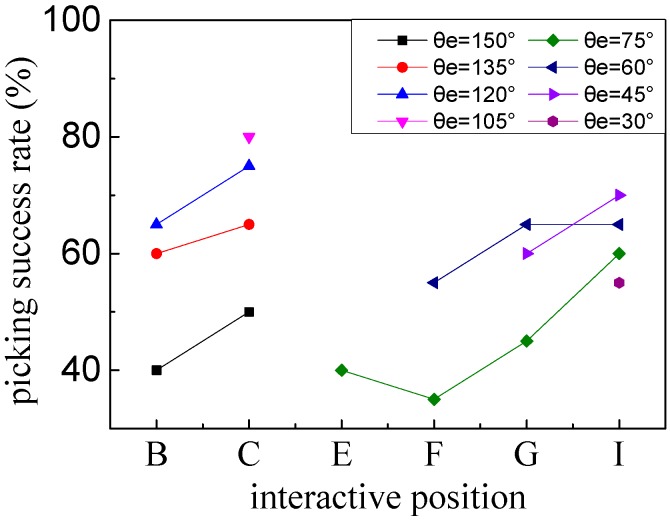
Effects of selected interactive positions with special tilt angles for valid picking of a microsphere under an e-beam irradiation time of 40 s.

After completely damaging the adhesive interface between the sphere and substrate, the effects of the selected interactive positions with special tilt angles for valid picking of a microsphere were described by continuing an e-beam irradiation time of 40 s with 10 trials in each group, as shown in Figure 8. The electrostatic force gathering carbonaceous contaminants under a vacuum environment could directly affect new adhesive interfaces between the tip and the sphere. It is obvious that the interactive positions at point *B* within a tilt angle of *θ_e_*, of approximately 120°–135°; point *C* within a tilt angle of *θ_e_*, of approximately 105°–120°; point *G* within a tilt angle of *θ_e_*, of about 45°–60°; and point *I* within a tilt angle of *θ_e_*, of about 30°–60° possess relatively higher picking success rates. A relatively higher picking success rate may be interpreted to be the result of new strong adhesive interfaces formed by effective e-beam irradiation onto the exposed contact area between the tip-sphere interface. In an extreme case, if assuming that the nonconductive microsphere is large enough, the selected interactive positions located in the upper part of the semi-sphere could have higher picking success rates.

### 3.3. Releasing Operation

**Figure 9:**
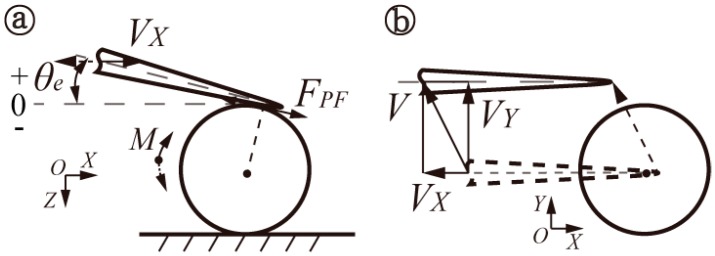
Releasing manipulations of a microsphere by effectively adjusting the end tilt angle (*θ_e_*) to easily destroy new tip-sphere adhesion layers.

When the sphere was picked up and then transported to the pre-appointed destination, it needed to overcome the adhesive forces existing in the new tip-sphere adhesion interface to achieve a valid release onto the substrate. As shown in Figure 9, the new adhesive layer was destroyed by adjusting the proper end tilt angle (*θ_e_*), before slow alternating repeated movements (*V_x_*) along the positive and reverse directions of *X* were used. This was achieved by determining whether the sphere generated relative rolling. Following this, a larger lateral moving speed (*V_y_*) along the *Y* direction was employed to accomplish release.

Here, due to a new adhesion layer formed by micro-scale forces, low friction factors between the sphere-substrate and tip-sphere interfaces were chosen as 0.3 and 0.1, respectively. Based on the theoretical analyses and operation conditions, an end tilt angle (*θ_e_*) of 5°–30° was utilized in the releasing experiments.

The new formation of a tip-sphere adhesion interface possessed minor adhesive forces due to nonconductive properties, so it seemed that it would be easily destroyed. Following this, the effective separation moving in the plane of *X*–*Y* would be possible. Considering the limited operation spaces, viewed in the experimental results, a smaller end tilt angle (*θ_e_*) within approximately 5°–15° could lead to a valid release of microspheres. According to the theoretical analysis, it can be deduced that releasing a conductive microsphere requires a relatively smaller end tilt angle compared to that of a nonconductive microsphere.

**Figure 10:**
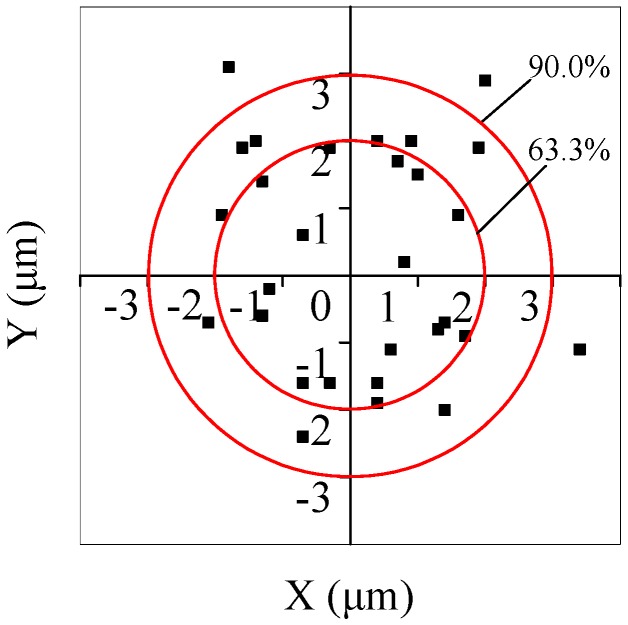
Figure illustrating the actual releasing landed positions, which deviated from the pre-setting reference point.

By means of relative rolling along the *X* direction to accomplish release, deviations of geometrical central points of the microsphere in the *X*–*Y* plane, compared with those of pre-setting landed locations, were utilized to weigh placement effects. Figure 10 indicates that the actual landed central positions deviated from the pre-setting reference point, where positive and negative values were representative of relative locations away from it. The experiments had 30 trials, while the releasing accuracy of deviation ranges within ±2 μm and ±3 μm were 63.3% and 90.0% along the *X* and *Y* axes, respectively. Therefore, it is quite clear that the releasing operation proposed can effectively accomplish a higher repeatability. In particular, the maximum deviation of the landed position was 3.4 μm, which seemed not to be allowed in pursuing an accurate placement operation. Thus, short transportation and irradiation times are required in order to avoid the large adhesion forces formed by contaminant deposition.

## 4. Conclusions

For depicting the interactive behaviors that exist in interactive contact, picking up, and placement of nonconductive microspheres, theoretical models were primarily classified and simplified to analyze the relationships between external and internal forces. Effective and reliable contact with a glass microsphere was shown, based on the combination of distinct alterations of light-shadow with relative movement displacement between the tip and sphere using a scanning electron microscope. Manipulation effects of the typical interactive positions and different end tilt angles when picking up microspheres were experimentally and quantitatively analyzed and discussed. Successful picking operations can be implemented using e-beam irradiation onto the proper interactive positions with end tilt angles. Finally, valid releasing operations were realized by adjusting the end tilt angles to damage the new tip-sphere adhesion layer. Furthermore, degrees of landed positions that deviated from the pre-set points were quantified.
